# Re-contact rates with a UK ambulance service following paramedic referral to a falls prevention service for those aged ≥ 65 years: a retrospective cohort study

**DOI:** 10.29045/14784726.2020.09.5.2.18

**Published:** 2020-09-01

**Authors:** Jamie Scott

**Affiliations:** Southern GP Federation Support Unit ORCID iD: https://orcid.org/0000-0003-2402-021X

**Keywords:** accidental falls, allied health personnel, referral and consultation

## Abstract

**Background:**

Falls in older populations constitute a large proportion of the workload for UK ambulance services, and cost the NHS over £2.3 billion per year. A large proportion of older fallers are not conveyed to an emergency department (ED), representing a vulnerable group of patients. New pathways have been developed for paramedics to refer this group directly to falls prevention services.

**Objectives:**

This study aimed to investigate the re-contact rates and characteristics of service users aged ≥ 65 years who fell and were referred to a falls prevention service by paramedics, and to describe the characteristics of those who re-contacted the ambulance service after referral.

**Methods:**

A retrospective cross-sectional cohort study was carried out in the geographical area covered by the South Eastern division of the Northern Ireland Ambulance Service (NIAS) between 1 July and 30 September 2017. The primary outcome was the rate of subsequent contacts with the ambulance service following referral.

**Results:**

There were 1079 incidents of falls in service users aged ≥ 65 years. A referral rate of 7% (n = 75) was reported, constituting the study cohort. Re-contact rates were 37.3% (n = 28) within 1 month and 70.7% (n = 53) within 6 months. Women and those exposed to a ‘long lie’ were more likely to re-contact, while those with cognitive impairment appeared particularly vulnerable to falls and repeat falls. Repeat falls were common. Documentation by attending clinicians was generally poor.

**Conclusion:**

Future research should investigate the efficacy of paramedic referral to falls prevention services. Interventions targeted at reducing long lies and investigating optimal interventions for those with cognitive impairments should also be explored. Improving clinical documentation will facilitate future research.

## Introduction

### Background

Falls are common in older populations, accounting for 8% of the workload of UK ambulance services (Snooks et al., 2006). The annual cost to the NHS is estimated to be in excess of £2.3 billion (NICE, 2018). High ambulance re-attendance rates in this population lead to increased operational costs for ambulance services (Simpson et al., 2014) that are likely to increase exponentially with a rapidly ageing population (Office for National Statistics, 2015).

The Prevention of Falls Network Europe (ProFaNE) defines a fall as ‘an unexpected event in which the participant comes to rest on the ground, floor, or lower level’ (Lamb et al., 2005, p. 1619). Often perceived as a low acuity callout for an emergency ambulance (Simpson et al., 2017), the physical and psychological impact of a fall on an individual can be life-changing. Reduced quality of life, social isolation, increased dependency, institutionalisation and increased mortality have all been linked with older fallers (Snooks et al., 2006).

Most falls in people aged ≥ 65 years occur at home, with 73% of fallers sustaining an injury (Simpson et al., 2014). Injuries range from bruises, lacerations and soft tissue injuries to fractures of the hip or spine, and the increased number of comorbidities and polypharmacy associated with older populations are also associated with poor outcomes (Aronsson et al., 2014; Boland et al., 2014; Wu et al., 2013). Injuries sustained from falls thus have the potential to be debilitating, and are associated with excess mortality rates (Kannegaard et al., 2010).

Until relatively recently, emergency ambulance crews would either transport older fallers to an ED or discharge them at scene. While attending crews assess the extent of injuries and the need for immediate assessment, they do not routinely assess or address underlying risk factors for falls (Logan et al., 2010). The 25–40% of older fallers not conveyed to an ED (Simpson et al., 2014; Snooks et al., 2006; Tiedemann et al., 2013) thus represent a high-risk group at increased risk of subsequent falls (Tiedemann et al., 2013; Wu et al., 2013), further emergency healthcare contact and increased mortality rates (Snooks et al., 2006).

Following a review of health and social care services in Northern Ireland (Compton, 2011), a regional falls prevention referral pathway is now available that enables paramedics to directly refer service users who have fallen at home – but who are not deemed to require immediate acute medical care – to the local Health and Social Care trust’s falls co-ordinator. These referrals are then triaged, and onward referral is made to consultant-led fall clinics and/or community fall prevention teams as appropriate. This grants this particularly vulnerable group access to a multifactorial fall prevention programme; however, no work has been undertaken to assess the efficacy of this initiative or its impact on the Northern Ireland Ambulance Service (NIAS) and its service users.

A recent UK-based large-scale cluster randomised trial concluded that multifactorial interventions for older fallers reduced the number of subsequent 999 calls (or ‘re-contacts’) made (Snooks et al., 2017), and a Cochrane review of interventions for preventing falls showed that these interventions reduce the rate of falls (Gillespie et al., 2012). However, it has also been suggested that these interventions do not reduce the risk of falling (Gillespie et al., 2012), attendance at ED or mortality rates (Snooks et al., 2017). The re-contact rates of the fall prevention pathway in Northern Ireland have not previously been studied.

Much of the previous research into older fallers excludes service users with cognitive impairments, due to self-report methodologies (Snooks et al., 2017; Tiedemann et al., 2013; Wu et al., 2013). There is also limited information on patients who experience an extended period of time on the floor following a fall, or a ‘long lie’ – a common complication for older fallers (Simpson et al., 2014) and an established risk factor for poor outcomes (Fleming & Brayne, 2008). As such, the current study aims to identify and investigate the demographics of sub-groups of patients with cognitive impairments, and those exposed to a long lie.

### Objectives

1. The primary objective is to find out the number of re-contacts made with the ambulance service at 1- and 6-month follow-up intervals by people aged ≥ 65 years who had been referred to a falls prevention service.
2. Investigate the characteristics and epidemiology of patients who re-contact the ambulance service following referral.
3. Identify sub-groups within the cohort, including those exposed to long lies and those with cognitive impairment, and explore patterns emerging from the data.

## Methods

### Study design

A retrospective cross-sectional cohort design was employed. This design was both appropriate to the research objectives and practicable given the timescale and resources available.

### Setting

The study took place in the area covered by the South Eastern division of the NIAS. This area is comprised of several heavily populated urban areas, as well as sparsely populated rural areas, and has an estimated population of 358,963, with just over 18% (n = 65,880) aged ≥ 65 years (the highest proportion of ≥ 65s of all NIAS divisions) (NISRA, 2018).

### Study size

During the study period, there were 1079 calls where the service user was aged ≥ 65 years and had the chief complaint of a fall. Seven per cent (n = 75) of these incidents resulted in referral to the falls prevention service, and this group constituted the study cohort.

### Participants

**Figure 1:**
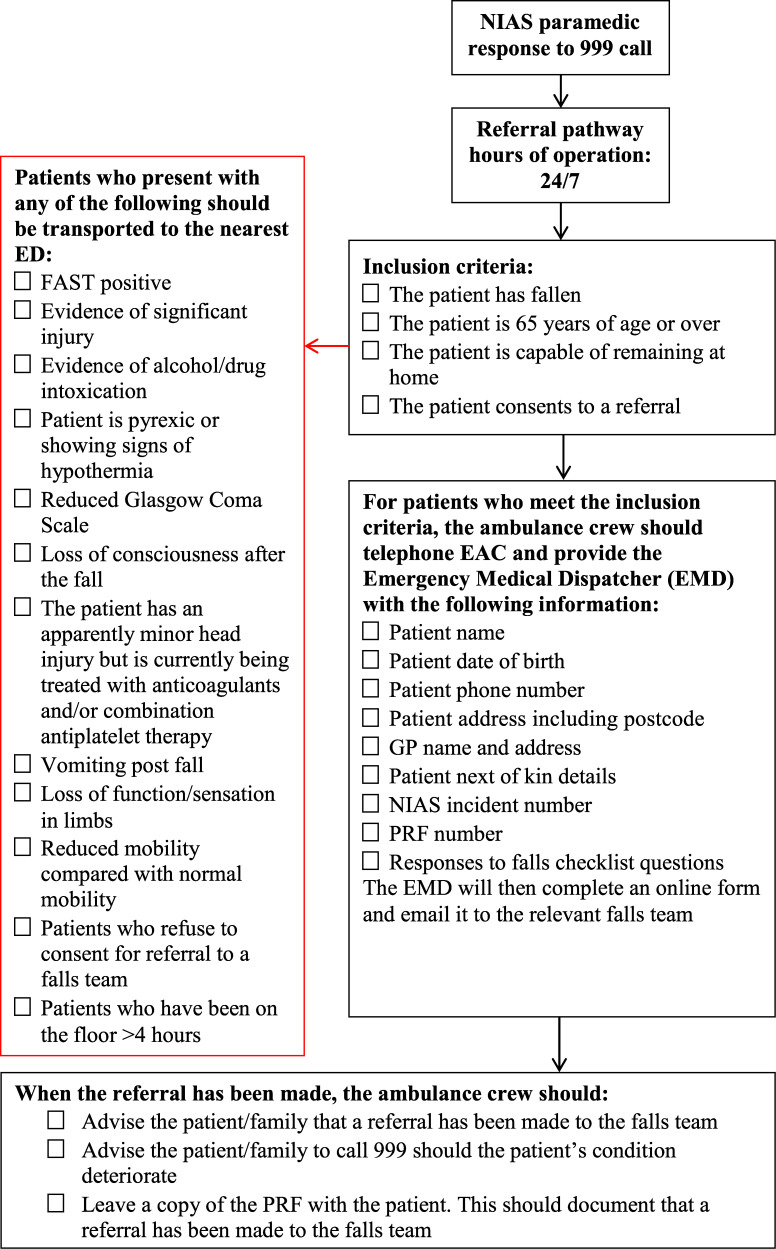
Figure 1. NIAS referral guidance for patients who have fallen.

Source: NIAS (2018).

All individuals aged ≥ 65 years in the geographical area covered by the South Eastern division of the NIAS who were attended by an NIAS paramedic for a fall and referred to the falls prevention service during the 3-month period from 1 July to 30 September 2017 were included. The NIAS referral protocol can be seen in Figure 1.

Participants were identified using NIAS Emergency Ambulance Control (EAC) call data by searching for incidents coded by the Advanced Medical Priority Dispatch System (AMPDS) as ‘falls’ and/or those whose chief complaint recorded on Patient Report Forms (PRFs) by the attending ambulance crew was a fall. Copies of the PRFs were then requested for each of these cases in order to gather and analyse patient outcomes and epidemiological and clinical data.

Re-contacts were identified by searching the EAC database for subsequent contacts with the NIAS within 1-month and 6-month follow-up intervals made by the cohort of 75 service users who were referred to the falls prevention service.

Initial identification of cases, and of their subsequent re-contacts with the ambulance service, was carried out by the NIAS Quality Improvement (QI) team who work with this data as part of their daily duties. This allowed records to be pseudonymised prior to being seen by the researcher.

### Data sources/measurement

Initial AMPDS coding of calls, and operational response data including call times, addresses and type of residence, were taken from the EAC database. Some epidemiological data (i.e. age and sex) were garnered from PRFs.

Outcomes were measured using PRF data and the EAC control database. Clinical data, including medical history, drug history, clinical observations, treatments and onset time of the fall, were collected from PRF data.

### Long lies

Time spent on the floor following a fall had to be calculated using existing data. For the purposes of this study, a long lie was assumed if ≥ 1 hour had passed between the recorded onset of the fall and the arrival of the ambulance. If this time was under 1 hour, then the patient was assumed not to have experienced a long lie.

### Bias

Bias was expected to be low, as the outcomes under study were not the original reason for which the data were collected (Mann, 2003). Descriptive studies are particularly vulnerable to selection bias (Gerhard, 2008). In this study, 100% of eligible service users within the timeframe were included in the analysis, and the aim of the study was to measure the prevalence of re-contact rates within the study cohort.

### Confounding variables

The study took place during mainly summer months, and the region chosen for the study was that with the highest proportion of its population aged over 65. These factors may have had a distorting effect on the results.

### Statistical methods

Descriptive statistics were used to summarise the epidemiology and characteristics of the cohort. The small sample size determined that the data were insufficiently powered to utilise inferential statistics. Although inferential statistics would have been desirable, descriptive statistics allow the objectives of this explorative study to be met, and it is hoped that the results will stimulate further research into the areas identified.

## Results

### Participants

**Figure 2:**
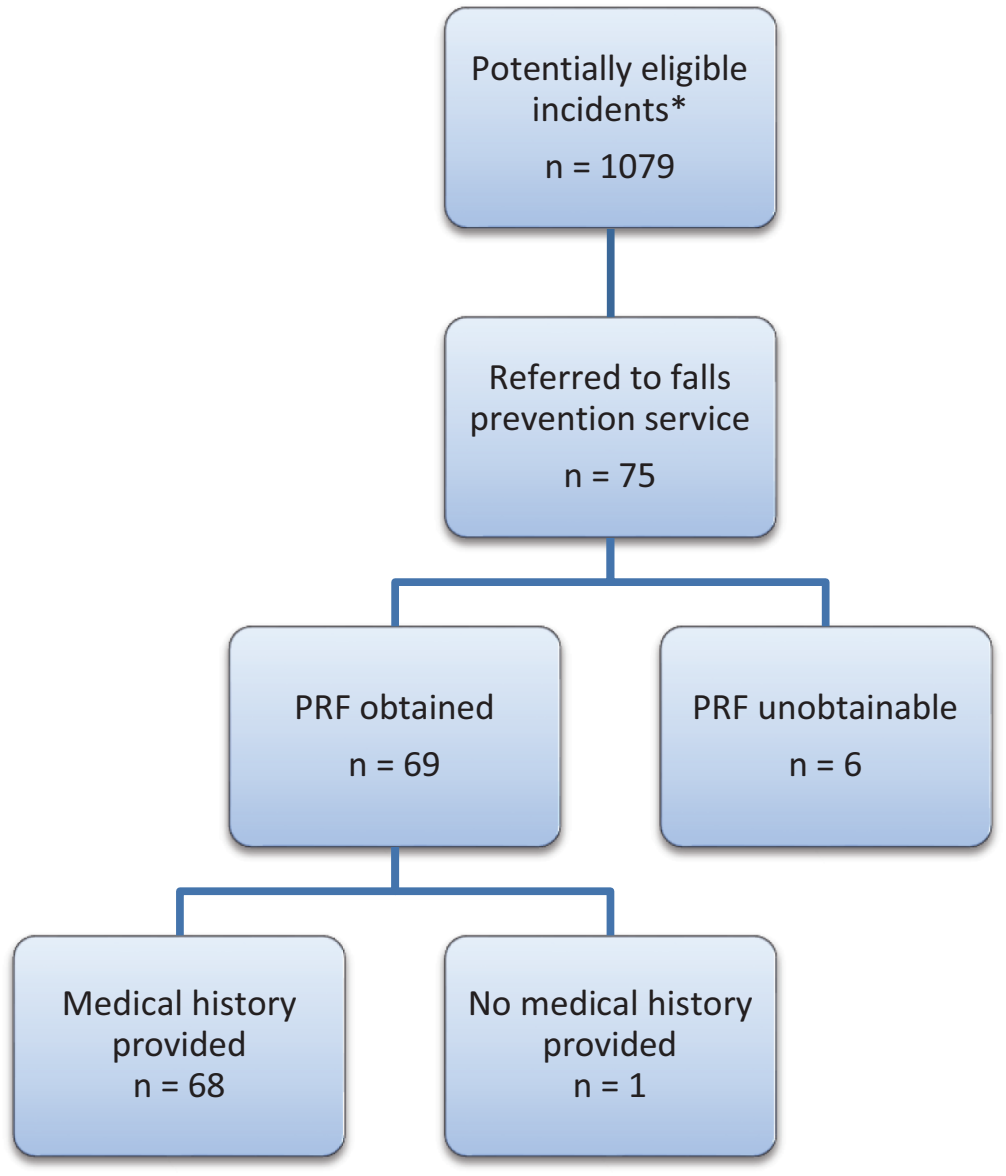
Figure 2. Flowchart showing participant numbers at each stage of data collection.

Note: * Incidents occurring during the study period where the patient was aged ≥ 65 and the chief complaint was a fall.

The number of individuals at each stage of data collection is summarised in Figure 2.

### Descriptive data

In the six cases where PRFs could not be obtained, re-contacts and some epidemiological information could be obtained but no clinical information was available for these service users. One individual declined to provide a medical history beyond a previous history of falls, so limited data were also available on this individual.

Descriptive data for the 75 participants included in analysis can be found in Table 1.

**Table 1.** Demographic data.

	**Referred to falls prevention service**	**Re-contacts within 6 months**
**Sex**	**n** = **75**	**n** = **136**
Male	36 (48%)	54 (39.7%)
Female	39 (52%)	82 (60.3%)
**Accommodation**	**n** = **75**	**n** = **136**
Private residence	60 (80%)	110 (80.9%)
Sheltered accommodation	11 (14.7%)	18 (13.2%)
Nursing/care home	4 (5.3%)	8 (5.9%)

### Primary outcome data

The cohort of 75 service users made 136 re-contacts within 6 months of referral to the falls prevention team. The number of re-contacts made within 1-month and 6-month windows are summarised in Table 2.

**Table 2.** Re-contacts in 1- and 6-month follow-up intervals.

	n	%
Re-contacted at least once within 1 month	28	37.3
Re-contacted at least once within 6 months	53	70.7
Re-contacted > once within 1 month	9	12
Re-contacted > once within 6 months	31	41.3
Re-contacted ≥ 5 times within 6 months	9	12
Did not re-contact within 6 months	22	29.3

### Demographics

The mean age of those who re-contacted at least once in the 6-month follow-up interval was 84.9 (SD: 9.07, range: 67–100). Other demographic information for those who re-contacted can be found in Table 1.

### Reason for re-contact

PRFs could not be obtained for 35 of the 136 re-contacts due to the PRF not being available, the call being passed to another service, the resource being cancelled or a resource not being allocated. This left 101 re-contacts for which PRF data were available for full analysis.

### Repeat falls

82.8% (n = 24) of patients with a history of falls documented on their PRF in their initial contact (n = 29) re-contacted the ambulance service at least once within the 6-month follow-up period.

Of the 101 re-contacts for which data were available, 58% (n = 59) were for further falls. Table 3 summarises the outcomes for these re-contacts. These re-contacts were made by a total of 37 individuals, representing a very interesting sub-group that merited further analysis.

**Table 3.** Outcomes for re-contacts for further falls.

Outcome (n = 59)	n	%
Emergency department	23	39
Re-referred to falls prevention team	15	25.4
Left at scene with no referral	11	18.6
Refused transport and referral to falls prevention service	4	6.8
Refused transport to emergency department	3	5.1
Refused referral to falls prevention service	2	3.4
Referred to out-of-hours GP	1	1.7

The mean age of this sub-group was 84.9 years (SD: 9.07; range: 67–100), and the mean number of days elapsed between the initial contact and the first re-contact with the ambulance service was 51.6 days (SD: 54.1, range: 0–180). Descriptive data for this sub-group can be found in Table 4.

**Table 4.** Demographic and clinical data for the sub-group who re-contacted for further falls.

Re-contacted for another fall within 6 months (n = 37)		
	**n**	**%**
**Sex**		
Male	13	35.1
Female	24	64.9
**Accommodation**		
Private residence	28	75.7
Sheltered accommodation	7	18.9
Nursing home	2	5.4
Re-contacted for another fall within 6 months and PRF obtained (n = 34)		
	**n**	**%**
History of falls	17	50
Re-contacted for another fall within 6 months, PRF obtained and patient provided medical history (n = 33)		
	**n**	**%**
Cardiac history	12	36.4
Cognitive impairment	10	30.3
Musculoskeletal history	9	37.3
Hypertension	7	21.2
Diabetes	6	18.2
Cerebrovascular attack (CVA) / Transient ischaemic attack (TIA)	5	15.2

### Long lies

81.2% (n = 56) of clinicians documented the onset time of the fall on the PRF, allowing time spent on the floor to be estimated. The mean time elapsed between the fall and the arrival of the ambulance for these 56 patients was 01:19:06 (SD: 01:41:10; range: 00:08:00–08:38:00). In 41.1% (n = 23) of these cases, an hour had elapsed between the time of the fall and the arrival of an ambulance. 78.2% (n = 18) of patients exposed to a long lie re-contacted at least once within 6 months. Comparatively, 60.6% (n = 20) of the 33 patients who waited less than an hour re-contacted in the same follow-up period.

### Cognitive impairment

The re-contact rate within the 6-month follow-up period for those with cognitive impairment (n = 20) was 85% (n = 17), while service users with a dementia diagnosis (n = 15) had a 6-month re-contact rate of 93.3% (n = 14). 30.3% (n = 10) of the 33 service users who re-contacted for further falls, and had clinical data available, had a documented history of cognitive impairment.

### Documentation

Although outside the scope of this study, it was interesting to note that in the 68 cases where PRFs were available, recording of medical history by the attending clinicians was generally poor and inconsistent. This merited further analysis, and documentation was thus benchmarked against NIAS clinical audit criteria for falls (see Table 5).

**Table 5.** Proportion of PRFs meeting NIAS clinical audit criteria.

Clinical criteria	Number of PRFs	Percentage of PRFs
Two timed sets of basic observations recorded	67	97.1
Blood glucose recorded	68	98.6
FAST test recorded	58	84.1
Cause of fall recorded	50	72.5
Mobility assessment documented	38	55.1
12-lead ECG recorded and interpreted	36	52.2
History of falls recorded	32	46.4
Worsening care advice given and recorded	29	42
**Documented all clinical audit criteria**	**3**	**4.3**
**Documented all clinical audit criteria except 12-lead ECG**	**5**	**7.2**

Documentation of past medical history and drug history was poor, making analysis of clinical characteristics impossible. No past medical or drug histories were recorded on many PRFs, instead referring to a list of medications, stating ‘known to the patient’ or leaving the area blank altogether.

21.7% (n = 15) of the cohort refused to travel to ED against the advice of the attending clinician, with a further 36.2% (n = 25) of PRFs stating that the patient ‘declined’ conveyance to ED, but not documenting a formal refusal to travel against advice. Thus, a total of 57.9% (n = 40) of the cohort declined/refused transport to an ED and were subsequently referred.

## Discussion

### Key results

Falls in older populations present a major challenge to ambulance services and to the health service in general. Research suggests that community-based multifactorial falls interventions are the most appropriate intervention (Gillespie et al., 2012), with this study reporting a referral rate of 7% to such a service. This is slightly lower than the 8.4% of patients referred in the intervention wing of the SAFER 2 trial, but considerably higher than the 1.1% referred by the control wing (Snooks et al., 2017). It should be noted that intervention-wing paramedics in the SAFER 2 trial received an additional training package, while control paramedics continued their usual practice (Snooks et al., 2017).

The cohort of 75 referred individuals led to 136 re-contacts with the ambulance service within the 6-month follow-up period, with an overall re-contact rate of 70.7% (n = 53) in this timeframe. This is only slightly lower than the 71.1% of patients who died or had a further emergency admission, ED attendance or 999 call in the SAFER 2 trial (Snooks et al., 2017). The re-contact rate reported by the current study only represents further ambulance contacts, while the SAFER 2 trial allowed for patients who may have died or had further ED attendances within the follow-up period; as such, the actual re-contact figure may have been higher.

A pattern of repeat falls and repeat ambulance usage emerged from this cohort. 70.7% of referred older fallers re-contacted the ambulance service at least once within 6 months, with 57.8% of these re-contacts for repeat falls. In addition, a large proportion of service users with a documented history of falls (82.8%) re-contacted the ambulance service at least once within the 6-month follow-up period. This is consistent with existing literature suggesting that a history of previous falls is predictive of future healthcare contacts (Simpson et al., 2014; Wu et al., 2013).

Women in this cohort were more likely to re-contact overall, and to re-contact for repeat falls, than men, adding weight to previous suggestions that female sex is predictive of repeat falls (Wu et al., 2013). Those who were exposed to long lies were also more likely to re-contact the ambulance service within the follow-up period, a finding consistent with previous work reporting poorer outcomes for this patient group (Fleming & Brayne, 2008).

Patients with cognitive impairments appeared particularly vulnerable to falls and repeat falls, an interesting finding as this population has been excluded from previous research due to its reliance on self-report methodology. This highlights this sub-group as worthy of further investigation, particularly research that aims to identify and test interventions specifically targeted at this group. Fleming and Brayne (2008) note that cognitive impairment was linked to long lies, and suggest automatic fall recognition technology as a potential intervention for both sub-groups.

### Documentation

Documentation by attending paramedics was generally poor. This can be partly attributed to the practical challenges that prehospital clinicians face in attaining an accurate medical, drug and social history in the absence of patient records (Halter et al., 2011). The NIAS’s planned move to electronic PRFs and remote access to patient records thus has the potential to improve documentation, decision making and care.

A noteworthy, yet anecdotal, observation was that documentation was suggestive of paramedics using the fall referral pathways as a safety net for individuals who decline ED attendance, rather than making a clinical decision not to transport. A culture of ambulance clinicians ‘covering their backs’ has previously been reported as a potential explanation for this (Comans et al., 2013; Halter et al., 2011).

### Limitations

There is a well-documented disparity between the ProFaNE definition of a fall (Lamb et al., 2005) and what AMPDS codes as a fall, with 33% of falls not coded as such by ambulance dispatch software (Snooks et al., 2011). This issue was partially addressed by cross-referencing the chief complaint recorded by the ambulance clinician with the code allocated by dispatch. However, there is also disparity between this definition and how ambulance clinicians may define a fall (Halter et al., 2011). Thus, some patients may have met the criteria for referral but not been referred.

It was not possible to accurately measure the incidence of long lies. As such, an assumption was made that the patient had been on the floor from the onset of the fall documented by the ambulance clinician until the arrival of the ambulance. In reality, some individuals may have been assisted off the floor prior to the arrival of an ambulance, and it is often difficult for ambulance crews to ascertain the exact time of a fall. In addition, no onset time was recorded for 18.8% (n = 13) of the cohort, meaning that time on the floor could not be calculated. However, the assumption allowed the researcher to investigate some of the potential impacts of a long lie and to recommend further research and potential interventions.

The setting for the current study included both urban and rural areas, with seven ambulance stations covering a population of 358,963 people. All eligible patients were included in the analysis. The results can therefore be generalised to other UK ambulance services covering similar geographical areas and to clinical staff with comparable levels of training and education.

Although the results of this investigation are useful in identifying trends in the data and areas for further study, caution must be used in drawing conclusions. The data may be used alongside existing literature to make inferences about the effectiveness of the pathway in question, but causation cannot be inferred due to the relatively low number of participants, the lack of a control group and the limitations discussed above.

## Conclusion

Future research is required to investigate the efficacy of paramedic referral to falls prevention services and to explore other alternatives to transport to ED. Experimental studies are also required that test the efficacy and operational costs of interventions to reduce incidence of long lies and investigate optimal interventions for older fallers with cognitive impairments.

Improvement of documentation, through innovations such as electronic patient report forms, will facilitate future work in this area.

## Acknowledgements

The author would like to thank Ciaran McKenna, Clinical Service Improvement lead at NIAS, Alison Vitty, Corporate Manager at NIAS, and the NIAS Quality Improvement team.

## Guarantor statement

JS acts as the guarantor for this article.

## Conflict of interest

None declared.

## Ethics

This research was granted ethical approval by the University of Cumbria’s Research Ethics Panel. The work was exempt from Research Ethics Committee (REC) review as raw data were handled by the NIAS QI team (who use the data as part of their normal daily duties) and were pseudonymised in line with General Data Protection Regulation (GDPR) guidance prior to being seen by the researcher (IRAS, 2020). A confidentiality agreement with NIAS was signed by the researcher.

## Funding

None.
